# Quality of life and health care consultation in 13 to 18 year olds with abdominal pain predominant functional gastrointestinal diseases

**DOI:** 10.1186/1471-230X-14-150

**Published:** 2014-08-21

**Authors:** Niranga Manjuri Devanarayana, Shaman Rajindrajith, Marc A Benninga

**Affiliations:** 1 Department of Physiology, Faculty of Medicine, University of Kelaniya, Thalagolla Road, 11010 Ragama, Sri Lanka; 2 Department of Paediatrics, Faculty of Medicine, University of Kelaniya, Thalagolla Road, 11010 Ragama, Sri Lanka; 3 Department of Pediatric Gastroenterology and Nutrition, Emma Children’s Hospital, Academic Medical Centre, Amsterdam, The Netherlands

**Keywords:** Pain abdomen, Child, Functional gastrointestinal disorder, Health care consultation, Quality of life, Teenage

## Abstract

**Background:**

Abdominal pain predominant functional gastrointestinal diseases (AP-FGD) are commonly seen in the paediatric age group. It has significant impact on daily activities of affected children. Main objective of this study was to assess the health related quality of life (HRQoL) in children with AP-FGD.

**Method:**

This was a cross sectional survey conducted in children aged 13–18 years, in four randomly selected schools in Western province of Sri Lanka. Data was collected using a previously validated, self-administered questionnaire. It had questions on symptoms, HRQoL and health care consultation. AP-FGD were diagnosed using Rome III criteria.

**Results:**

A total of 1850 questionnaires were included in the analysis [males 1000 (54.1%), mean age 14.4 years and SD 1.3 years]. Of them, 305 (16.5%) had AP-FGD [irritable bowel syndrome = 91(4.9%), functional dyspepsia = 11 (0.6%), abdominal migraine = 37 (1.9%) and functional abdominal pain = 180 (9.7%)]. Lower HRQoL scores for physical (83.6 vs. 91.4 in controls), social (85.0 vs. 92.7), emotional (73.6 vs. 82.7) and school (75.0 vs. 82.5) functioning domains, and lower overall scores (79.6 vs. 88.0) were seen in children with AP-FGD (*p* < 0.001). A weak but significant negative correlation was observed between HRQoL score and severity of abdominal pain (r = −0.24, *p* < 0.0001). Eighty five children (27.9%) had sought healthcare for AP-FGD. Factors determining healthcare seeking were presence of abdominal bloating and vomiting (*p* < 0.05).

**Conclusions:**

Children with AP-FGD have lower quality of life in all 4 domains. Those with severe symptoms have lower HRQoL. Approximately 28% of children with AP-FGD seek healthcare for their symptoms.

## Background

The World Health Organization defines health as “a state of complete physical, mental and social well-being and not merely the absence of disease or infirmity” [1]. Quality of life is a term used to refer to an individual’s total well being. This includes all emotional, social and physical aspects of an individual’s life. When the phrase is used in reference to medicine, it is called as “Health Related quality of Life (HRQoL).

Abdominal pain predominant functional gastrointestinal diseases (AP-FGD) such as irritable bowel syndrome (IBS), functional abdominal pain (FAP), abdominal migraine (AM) and functional dyspepsia (FD), are seen in approximately 12% of children and adolescents [2,3]. Even though not life threatening, AP-FGD are chronic, troublesome disorders which can have significant impact on life of the affected children. In the absence of biological measures of disease activity, HRQoL becomes an important objective measure of health status in children suffering from AP-FGD.

Several studies have so far assessed the quality of life in children and adolescents with AP-FGD and all of them have reported lower quality of life [4-9]. Most studies were conducted in young children and have assessed HRQoL using a parent report form and include children of younger age groups [4-7]. Studies reporting quality of life in teenagers with AP-FGD are rare [8,9].

Abdominal pain is often an alarming symptom which leads to frequent healthcare consultation. Few studies conducted in children with abdominal pain, have reported healthcare consultation of 39 to 93% in affected children [10-14]. To date, no studies are available that have evaluated healthcare consultation in teenagers with AP-FGD.

HRQoL and healthcare seeking behaviours are likely to vary from country to country, community to community, depending on demographic and socio-cultural factors. HRQoL and healthcare consultation pattern in teenager are likely to be different from that of younger children. So far, no studies have assessed HRQoL and healthcare consultation in Sri Lankan teenagers with AP-FGD. This study was conducted with the main objective of assessing HRQoL and healthcare consultation in children aged 13–18 years in Sri Lanka and factors associated with them.

## Methods

This was a cross sectional survey conducted in in the Western province of Sri Lanka.

### Data collection

Western province of Sri Lanka has 1333 functioning government schools (similar to public schools). Of them, 427 are schools with students aged 13–18 years. From the list of these 427 schools available at the Provincial Education Office, four mixed gender schools were randomly selected by drawing lots. All children aged 13 to 18 years in these schools were invited to take part in the study.

Data on socio-demographic and family characteristics, symptoms, HRQoL and healthcare consultation were collected using a validated, self-administered questionnaire. Questionnaire was in native language (Sinhala). It consisted of 4 parts. First part contained questions on socio-demographic and family characteristics. Information regarding AP-FGD was collected using Rome III questionnaire for FGD (child report form for children above 10 years) (Part 2) [15]. This part of the questionnaire has been translated, validated and used for Sri Lankan children previously [2,3]. Part 3 was PedsQL, Pediatric Quality of Life Inventory 4.0 (Generic Core Scales) self report form for teens [16-18], which has been previously translated in to native language (Sinhala) and has undergone linguistic validation by Mapi Research Trust. The investigators have obtained permission to use this questionnaire for this study. Part 4 of the questionnaire contained questions regarding healthcare consultation. This part of the questionnaire has been developed by the investigators, pretested, and used previously in a school based study in Sri Lankan children [19].

This was an anonymous questionnaire, administered in examination setting, to ensure confidentiality and privacy. Research assistants were present and support was given during filling the questionnaire. Questionnaires were collected on the same day. Consent was obtained from school administration, teachers, parents and children themselves before administration of the questionnaire.

### Scales used

The HRQoL inventory consisted of 23 items. It was used to assess the physical functioning (8 items), emotional functioning (5 items), social functioning (5 items), and school functioning (5 items) of the child. A 5-point response scale is used (0 = never a problem; 1 = almost never a problem, 2 = sometimes a problem, 3 = often a problem, 4 = almost always a problem) to record the responses. Items were reverse scored and linearly transformed to a zero to 100 scale (0 = 100, 1 = 75, 2 = 50, 3 = 25, 4 = 0). Final HRQoL scores were computed out of 100, with higher scores indicating better HRQoL.

Symptom severity of abdominal pain, dyspepsia and bowel symptoms were recorded using a visual analogue scale (100 mm) rating between 0% to 100%, where 0% is not having symptoms at all and 100% is having very severe symptoms.

### Definitions used

AP-FGD; irritable bowel syndrome (IBS), functional dyspepsia (FD), abdominal migraine (AM) and functional abdominal pain (FAP), were diagnosed using Rome III criteria defined by Rasquin *et al.* in 2006 [20].

A child who has received treatment for abdominal pain during previous 3 months was considered as a healthcare consulter.

### Ethical approval

Ethical approval was obtained from the Ethical Review Committee of the Sri Lanka College of Paediatricians.

### Statistical analysis

The data were analyzed using EpiInfo (EpiInfo 6, version 6.04 (1996), Centres of Disease Control and Prevention, Atlanta, Georgia, USA and World Health Organization, Geneva, Switzerland). Total HRQoL scores were compared using unpaired *t*-test. Healthcare consultation between patients and controls were compared using *X*^2^ test. Multiple logistic regression analysis was used to evaluate independent association between factors identified as significant in the univariable analysis. All correlations were done using Pearson correlation coefficient. *p* < 0.05 was considered as significant.

## Results

A total of 1855 questionnaires were distributed and all of them were returned. Of them, 1850 (99.7%) properly filled questionnaires were included in the analysis. There were 1000 (54.1%) boys. Mean age of the participants was 14.4 years (SD 1.3 years).

A total of 305 (16.5%) of children had AP-FGD. IBS was seen in 91 (4.9%), FD was seen in 11 (0.6%), AM was seen in 37 (1.9%) and FAP was seen in 180 (9.7%). Of them 13 had both IBS and AM and 1 had AM and FD. AP-FGD were significantly more prevalent in girls [175 (20.1%) vs. 130 (13.0%) in boys, *p* < 0.0001)]. During analysis, 1545 children without AP-FGD were considered as controls.

### HRQoL in children with AP-FGD

Table 1 shows the mean HRQoL scores in children with all four types of AP-FGD and controls. Children with AP-FGD had lower HRQoL scores than controls in all 4 domains (physical, emotional, social and school functioning). When HRQoL scores of children with different types of AP-FGD were analysed, children with IBS and AM had lower HRQoL scores for all four domains, compared to controls. Those with FAP had lower HRQoL scores only for physical and emotional functioning domains. There was no statistical difference between children with FD and controls (Table 1).

**Table 1:** Health related quality of life (HRQoL) scores in children with abdominal predominant functional gastrointestinal diseases and controls

**Quality of life domains**	**Irritable bowel syndrome**	**Functional dyspepsia**	**Abdominal migraine**	**Functional abdominal pain**	**AP-FGD total**	**Controls**
	**Mean (SD)**	**Mean (SD)**	**Mean (SD)**	**Mean (SD)**	**Mean (SD)**	**Mean (SD)**
Physical functioning (%)	84.7 (15.1)****	94.0 (7.8)	81.6 (15.2)****	89.1 (12.1)***	87.9 (13.4)****	91.5 (10.9)
Emotional functioning (%)	70.1 (21.2)****	89.5 (9.3)	68.4 (22.8)****	77.8 (19.7)****	75.6 (20.3)****	83.4 (17.0)
Social functioning (%)	85.7 (16.5)****	95.0 (9.2)	86.3 (15.6)****	92.1 (12.1)	90.0 (14.0)****	92.7 (11.6)
School functioning (%)	74.1 (18.7)****	89.5 (12.5)	72.5 (19.3)****	79.9 (16.6)*	78.0 (18.0)****	82.6 (16.8)
Total HRQoL score (%)	79.6 (13.6)****	92.3 (5.6)	78.6 (12.7)****	85.6 (10.4)**	83.8 (11.8)****	88.1 (10.9)

AP-FGD = abdominal pain predominant functional gastrointestinal diseases.

*p = 0. 04, **p = 0.006, ***p = 0.003, **** *p* < 0.0001 compared to controls, unpaired *t*-test.

When HRQoL scores were compared between different AP-FGD types, lowest HRQoL scores were observed in children with AM (78.6%) and IBS (79.6%) (*p* < 0.001, compared to FAP and FD) (Table 1).

### Healthcare consultation in children with AP-FGD

Table 2 shows the percentage of healthcare consultation according to AP-FGD type. Healthcare consultation in patients with AP-FGD was 27.9%. In addition, 8.3% of controls have sought medical advice for abdominal pain due to other causes. When healthcare consultation between different AP-FGD types was compared, the highest rate was observed in children with AM (40.5%).

**Table 2:** Health care consultation in children with abdominal pain predominant functional gastrointestinal disorders

	**Health care consultation**	
	**Consulters**	**Non-consulters**
	***n *** **(%)**	***n *** **(%)**
**Irritable bowel syndrome**	27 (29.7%)	64 (70.3%)
**Functional dyspepsia**	4 (36.4%)	7 (63.6%)
**Abdominal migraine**	15 (40.5%)	22 (59.5%)
**Functional abdominal pain**	44 (24.4%)	136 (75.6%)
**Abdominal pain predominant FGD total**	85 (27.9%)	220 (72.1%)
**Controls**	129 (8.3%)	1416 (91.7%)

FGD = functional gastrointestinal disease.

### Factors affecting HRQoL in children with AP-FGD

As depicted in Table 3, no significant differences were found in scores obtained for HRQoL according to socio-demographic and family characteristics in children with AP-FGD (*p* > 0.05).

**Table 3:** Quality of life scores and heath care consultation in children with abdominal pain predominant functional gastrointestinal disorders according to the socio-demongraphic and family characteristics

**Variable**	**Health related quality of life (%)**	**Health care consultation**
	**Mean (SD)**	***n *** **(%)**
***Age***		
13 years	86.5 (10.2)	26 (35.1%)
14 years	84.5 (12.7)	18 (22.8%)
15 years	82.1 (11.0)	22 (31.0%)
16 years	81.9 (11.9)	7 (15.9%)
17 years	82.3 (13.7)	8 (38.1%)
18 years	81.6 (11.8)	4 (25.0%)
***Sex***		
Male	84.7 (12.4)	40 (30.8%)
Female	83.1 (11.4)	45 (25.7%)
***Family size***		
Only child	86.2 (11.2)	11 (37.9%)
2 children	84.2 (11.7)	42 (28.0%)
3 children	83.3 (11.5)	27 (27.6%)
4 children	84.7 (11.2)	4 (19.0%)
5 or more children	67.4 (17.4)	1 (14.3%)
***Birth order***		
1st	83.4 (12.5)	45 (30.2%)
2nd	84.5 (10.9)	31 (28.7%)
3rd	83.7 (9.4)	5 (14.3%)
4th	85.1 (12.9)	3 (30.0%)
5th or more	68.1 (24.3)	1 (33.3%)
***Father’s social class***		
Leading profession (e.g. doctor, engineer)	84.7 (12.3)	15 (33.3%)
Lesser profession (e.g. nurse, teacher)	83.7 (12.2)	6 (31.6%)
Skilled non-manual (e.g. clerk)	85.4 (13.3)	9 (24.3%)
Skilled manual (e.g. mason, carpenter)	83.2 (11.8)	36 (27.7%)
Unskilled/unemployed	82.4 (10.8)	11 (28.2%)
***Maternal employment***		
Leading profession (e.g. doctor, engineer)	83.6 (10.6)	2 (25.0%)
Lesser profession (e.g. nurse, teacher)	82.8 (15.8)	4 (28.6%)
Skilled non-manual (e.g. clerk)	84.0 (11.2)	2 (25.0%)
Skilled manual (e.g. mason, carpenter)	85.2 (11.3)	8 (22.9%)
Unskilled/unemployed	83.6 (11.8)	62 (25.8%)

HRQoL score had a weak but significant negative correlations with scores obtained for severity of abdominal pain (r = −0.24, 95% confidence interval (CI) -0.34 to −0.13, *p* < 0.0001), frequency of abdominal pain (r = −0.15, 95% CI −0.26 to −0.04, *p* = 0.009), severity of dyspepsia (r = −0.19, 95% CI −0.30 to −0.08, *p* = 0.001) and severity of bowel symptoms (r = −0.15, 95% CI −0.25 to 0.03, *p* = 0.01).

### Factors determining healthcare consultation in children with AP-FGD

**Figure 1:**
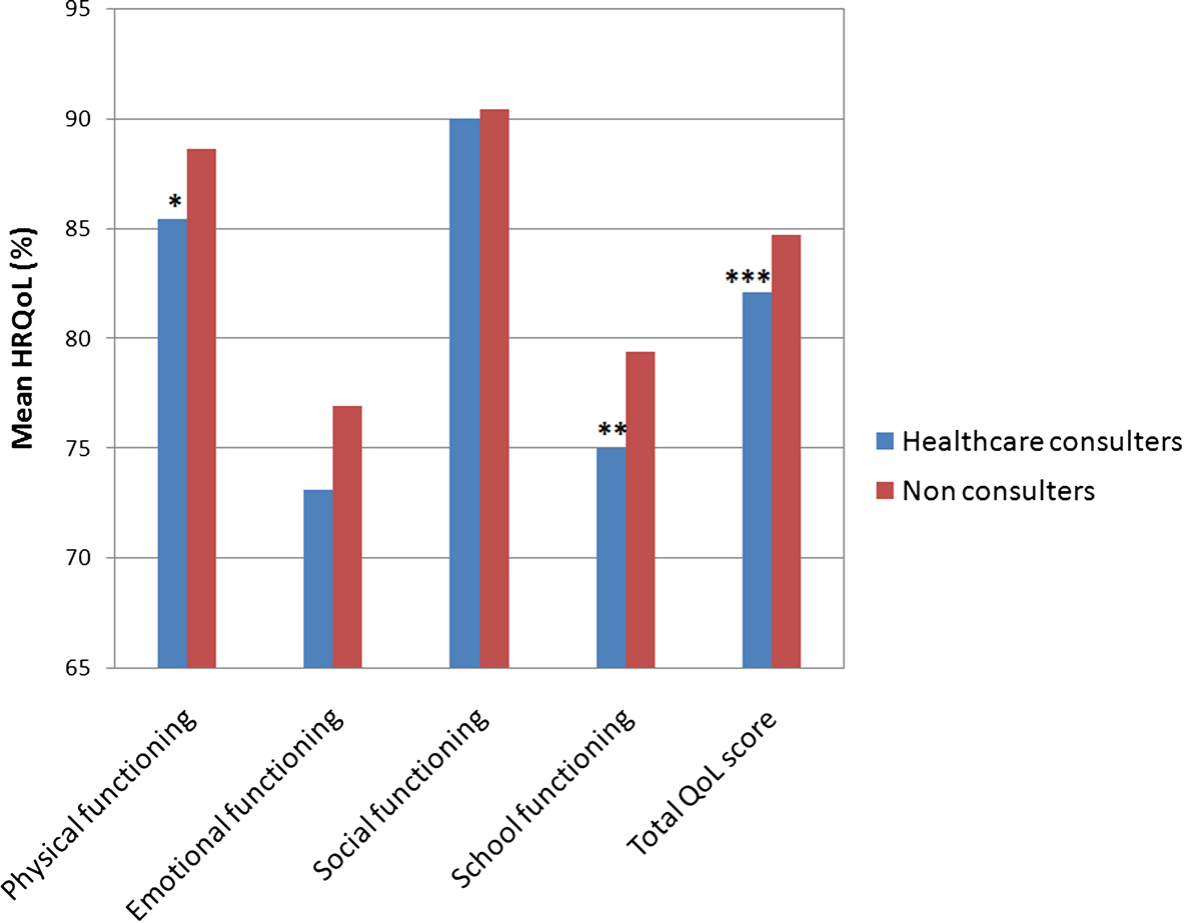
**Association between HRQOL and healthcare consultation.** *p = 0.03, **p = 0.048, ***p = 0.055, comparison between healthcare consulters and non consulters (unpaired *t*-test).

The association between socio-demographic factors and healthcare consultation is shown in Table 3. Table 4 shows the association between symptom characteristics and healthcare consultation. Following multiple logistic regression analysis, abdominal bloating [adjusted odds ratio (OR) 2.1, *p* = 0.04] and vomiting (adjusted OR 2.5, *p* = 0.02) remained to be significantly associated with healthcare consultation.In teenagers with AP-FGD, healthcare consulters had significantly higher scores for school functioning and physical functioning domains of HRQoL than non-consulters (Figure 1).

**Table 4:** Health care consultation in children with abdominal pain predominant functional gastrointestinal diseases according to symptoms

	**Health care consulters**	**Non consulters**	**Odd ratio (95% Confidence Interval)**	***P *** value*
	***n *** **(%)**	***n *** **(%)**		
**Frequency of abdominal pain**				
Once per week	61 (26.4%)	170 (73.6%)	0.8 (0.4-1.4)	0.32
Several times per week	20 (32.8%)	41 (67.2%)	1.3 (0.7-2.6)	0.34
Everyday	4 (30.8%)	9 (69.2%)	1.2 (0.3-4.3)	0.81
**Duration of a pain episodes**				
Less than 1 hour	41 (27.9%)	106 (72.1%)	1.0 (0.6-1.7)	0.99
1-2 hours	25 (34.2%)	48 (65.8%)	1.5 (0.8-2.7)	0.16
3- 4 hours	2 (11.8%)	15 (88.2%)	0.3 (0.1-1.6)	0.13
Most of the day	17 (25.0%)	51 (75.0%)	0.8 (0.4-1.6)	0.55
**Severity of pain**				
Mild	14 (26.4%)	39 (73.6%)	0.9 (0.4-1.9)	0.80
Moderate	43 (25.4%)	126 (74.6%)	0.8 (0.5-1.3)	0.29
Severe	28 (33.7%)	55 (66.3%)	1.5 (0.8-2.6)	0.20
**Location of pain**				
Upper abdomen	5 (38.5%)	8 (61.5%)	1.7 (0.5-5.8)	0.38
Periumbilical	58 (33.1%)	117 (66.9%)	1.9 (1.1-3.3)	0.02
Lower abdomen	8 (10.1%)	71 (89.9%)	0.2 (0.1-0.5)	<0.0001
Other	14 (36.8%)	24 (63.2%)	1.6 (0.7-3.5)	0.19
**Duration of the disease**				
2 months	26 (32.9%)	53 (67.1%)	1.4 (0.8-2.5)	0.25
3 months	18 (30.5%)	41 (69.5%)	1.2 (0.6-2.3)	0.61
4-11 months	12 (31.6%)	26 (68.4%)	1.2 (0.6-2.7)	0.58
More than 12 months	29 (22.5%)	100 (77.5%)	0.6 (0.4-1.1)	0.07
**Bloating**				
Yes	18 (43.9%)	23 (56.1%)	2.3 (1.1-4.8)	0.01
No	67 (25.4%)	197 (74.6%)		
**Early satiety**				
Yes	14 (31.1%)	31 (68.9%)	1.2 (0.6-2.5)	0.60
No	71 (27.3%)	189 (72.7%)		
**Loss of appetite**				
Yes	33 (30.3%)	76 (69.7%)	1.2 (0.7-2.1)	0.48
No	52 (26.5%)	144 (73.5%)		
**Nausea**				
Yes	36 (37.5%)	60 (62.5%)	2.0 (1.1-3.4)	0.01
No	49 (26.7%)	160 (77.3%)		
**Vomiting**				
Yes	18 (48.6%)	19 (51.4%)	2.8 (1.3-6.1)	0.002
No	67 (25.0%)	201 (75.0%)		
**Constipation**				
Yes	29 (37.2%)	49 (62.8%)	1.8 (1.0-3.2)	0.03
No	56 (24.7%)	171 (75.3%)		
**Loose stools**				
Yes	20 (34.5%)	38 (65.5%)	1.5 (0.8-2.8)	0.21
No	65 (26.3%)	182 (73.7%)		
**Sleep disturbance**				
Yes	32 (31.1%)	71 (68.9%)	1.3 (0.7-2.2)	0.37
No	53 (26.2%)	149 (73.8%)		
**School absenteeism**				
Yes	21 (32.3%)	44 (67.7%)	1.3 (0.7-2.5)	0.37
No	64 (26.7%)	176 (73.3%)		
**Disturbance in daily activities**				
Yes	43 (30.7%)	97 (69.3%)	1.3 (0.8-2.2)	0.31
No	42 (25.5%)	123 (74.5%)		
**Headache**				
Yes	38 (29.7%)	90 (70.3%)	1.2 (0.7-2.0)	0.31
No	47 (25.4%)	130 (74.6%)		
**Photophobia**				
Yes	16 (31.4%)	35 (68.6%)	1.2 (0.6-2.5)	0.54
No	69 (27.2%)	185 (72.8%)		
**Pallor**				
Yes	6 (23.1%)	20 (76.9%)	0.8 (0.3-2.1)	0.57
No	79 (28.3%)	200 (71.7%)		

*Chi-square test.

## Discussion

In this study, teenagers with AP-FGD had significantly lower HRQoL in all four domains; physical, emotional, social and school functioning. This lower HRQoL scores were significant in IBS, AM and FAP. Approximately 28% of affected children seek healthcare for their symptoms. Factors independently associated with healthcare consultation were abdominal bloating and vomiting.

In this study, we found a slightly higher prevalence of AP-FGD than previously reported in Sri Lanka. In a previous study conducted in children age 12–16 years in a semi-urban school, AP-FGD were seen in 13.8% [2]. Another study conducted in children aged 10–16 years in 8 schools of 4 provinces (out of 9 provinces of the country) has reported a prevalence of 12.5% [3]. A Colombian study conducted in a younger group of children (mean age 10 years) has reported AP-FGD in 10.8%. Differences between age groups and socio-geographical factors may have accounted for these differences in prevalence [21]. Contrast to previous studies, commonest AP-FGD in our teenagers was FAP not IBS [2,3]. Prevalence of FD is lower in the current study than previously reported in Sri Lanka (3.5%, 2.5%) and Colombia (1.7%) [2,3,21]. At the same time, we observed a significantly higher prevalence of FAP than in previous studies (9.7% in current study vs. 3.0%, 4.5% and 2.7% in previous studies) [2,3,21]. The exact reason for this is not clear, but might be due to differences in age groups.

Very few studies have evaluated HRQoL in teenagers with AP-FGD. A recent school based study, conducted in 10 to 17 years old children, has reported significantly lower quality of school work in children with IBS, aerophagia and cyclic vomiting [8]. Another study conducted in high school children in Korea has also reported similar results [9]. Lower HRQoL has also been reported in younger children with AP-FGD. Varni et al. have evaluated HRQoL using a generic score scale in children 2 to 18 years with IBS and reported lower scores in all 4 domains [5]. In another study, children with FAP (mean age 11.2 years) had significantly lower HRQoL in physical and emotional domains compared to healthy controls [4]. In that study, HRQoL scores in children with FAP were similar to those with chronic organic diseases such as gastro-oesophageal reflux disease and inflammatory bowel disease. Several studies conducted in preschool children and adults with AP-FGD have also reported lower quality of life in affected children and adults [6,7,22-25].

We have compared HRQoL between four different types of AP-FGD. The lowest score was observed in those with AM. Prolonged periods of severe abdominal pain, and presence of other troublesome symptoms such as headache, may have contributed to this lower HRQoL. In addition, children with IBS had HRQoL significantly lower than that of those with FAP and FD. Presence of bowel symptoms, in addition to abdominal pain, may have contributed to this finding. A recent study assessing school related quality of life in children 10 to 17 years reported lowest scores in those with FAP (9.0) followed by FD (10.5), IBS (11.3) and AM (11.6) [8]. This is different from scores obtained for school functioning in our study (Table 1). The fact that the previous study has used different scale and scoring system to measure school related quality of life and the differences in ages of children recruited and socio-cultural environments may have contributed to this difference.

We observed a weak, but significant inverse relationship between severity of symptoms (severity of abdominal pain, dyspepsia and bowel symptoms, and frequency of abdominal pain) and scores obtained for HRQoL. Similar to our results, Oostenbrink and co-workers have reported significant negative correlation between severity of abdominal pain and quality of life, in preschool children in the Netherlands [6]. Another study conducted in children with defecation disorders have found a similar correlation between HRQoL and abdominal pain and bloating [26]. Previous studies conducted in adult patients with functional gastrointestinal diseases have also reported lower HRQoL in patients with more severe symptoms [22]. There is a wide individual variation in perception of symptoms including pain. Sometimes patients with severe pain have fairly good quality of life while others with mild pain have poor quality of life. In our view, these individual variations may have contributed to the weak correlation observed in the current study between symptoms and HRQoL scores.

In this study, we did not find a relationship between HRQoL and age, gender, social class, maternal employment, family size and birth order. The relationship between socio-demographic and family characteristics and HRQoL has not been evaluated in paediatric patients with AP-FGD. However, contrary to our results, studies conducted in adult patients with functional gastrointestinal disorders have reported lower HRQoL in females compared to males [22].

In our study approximately 28% of affected children have sought medical advice for abdominal pain during previous 3 months. In addition, 8.3% of controls have also sought medical advice for abdominal pain due to other causes. There are no studies conducted in teenagers with AP-FGD on healthcare consultation. However, percentage of healthcare consultation in the current study is significantly lower than that reported in children with recurrent abdominal pain aged 5–15 years in Sri Lanka (70%) [12], and 9–15 years in Malaysia (45-48%) [10,11]. Another study conducted in German children has shown healthcare consultation of 52% in children (3–10 years) and 39% in adolescents (11–17 years) [13]. Some studies have reported healthcare use as high as 93% in children aged 4 to 17 years with non-specific abdominal pain [14]. Age groups of children included in those previous studies are lower than the teenagers we recruited in the current study. Generally, parents are more aware of the gastrointestinal symptoms and bowel habits of younger children and more worried about such symptoms when their children are younger. Therefore, younger children are more likely to seek healthcare than older children. This may have contributed to the higher prevalence of healthcare consultation seen in previous studies.

We expected higher healthcare consultations in children from higher social class, small families and those with severe symptoms and disturbances in day to day life. However, none of the other symptoms or socio-demographic and family characteristics were associated with healthcare consultation. Sri Lanka has well established government hospitals and clinics where healthcare is provided free of charge. Average distance from a home to a healthcare facility is approximately 1.4 km. This may have accounted for the lack of association between healthcare consultation and socio-economic factors. Previous studies conducted in children with abdominal pain have also failed to show an association between socioeconomic factors and healthcare consultation [10-12].

The symptoms independently associated with healthcare consultation in our study were abdominal bloating and vomiting. Similar to the current study, in the previous Sri Lankan study conducted in children aged 5 to 15 years with recurrent abdominal pain, the only symptom associated with healthcare consultation was vomiting [12]. In contrast to this, other previous studies conducted in younger children have shown significant associations between health care consultation and age of onset, severity, frequency and duration of pain episodes, school absenteeism, sleep interruption and disruption of normal activity [10,11,27]. It is parents who take the children to see a doctor. Unlike younger children, teenagers are reluctant to discuss their bodily symptoms with the parents. Some of the parents may not be aware of these symptoms in their children. That may be a reason for lack of association between healthcare consultation and some symptoms. However, a symptom like vomiting and bloating are visible to the parents and readily recognised. They are also alarming symptoms, especially in teenage girls in reproductive age. So those with bloating and vomiting are more likely to be taken to a doctor. In addition, due to variation in the perception of symptoms, the impact on the quality of life is more likely to influence healthcare consultation than the exact severity. In agreement with this, we found significantly lower scores for school functioning and physical functioning domains of HRQoL in healthcare consulters than in non consulters.

HRQoL is an indicator of the impact of a disease on the life of an individual and an indirect indicator of the disease severity. In this study we evaluated the impact of AP-FGD on physical, social, emotional and school functions of teenagers. Thirteen to eighteen years of life is a period with rapid physical, social and emotional development, and also a critical period in school education. Undesirable effects during this period are likely to have significant impact on development of the affected children and future social, emotional and financial stability. Long term and recurrent nature of the symptoms of AP-FGD and significantly decrease HRQoL of affected children are likely to have long term negative effects on their life. In addition, our results indicate that approximately quarter of Sri Lankan children with AP-FGD has sought medical advice for their symptoms during previous 3 months. Considering the high prevalence of this disease, AP-FGD in Sri Lankan teenagers are a significant burden on the already over-stretched healthcare system of the country. This needs to be taken in to consideration by healthcare personals, especially those looking after children with abdominal pain predominant functional gastrointestinal diseases. Prompt and effective management would not only decrease the suffering of the affected children, but also reduce the short term and long term impact of the disease on their life, their families, as well as the society.

The main strengths of the current study are inclusion of large number of teenagers and using standard and validated questionnaires for data collection. We believe that these have increased the reliability of our data. However, there were few limitations in this study. First, we did not investigate children to exclude organic causes for abdominal pain in the current questionnaire based survey. In a previous study we identified organic diseases in 10.9% of children with recurrent abdominal and nearly 89% had functional gastrointestinal diseases [28]. Similar results have been reported from other countries as well [29-31]. The organic diseases observed in the previous Sri Lankan study were urinary tract infection, gastroesophageal reflux disease, urinary calculi, antral gastritis, and intestinal amoebiasis [28]. Parasitic infestations such as giardiasis and amoebiasis have been considered to be possible mimickers of FGD; however, in that study, prevalence of these diseases was 1.8%, similar to several previous studies conducted in Sri Lanka [32]. Secondly, because this is self-administered questionnaire there may be some degree of recall bias. Thirdly, we assess healthcare consultation for abdominal pain not specifically for AP-FGD.

## Conclusions

This study has assessed HRQoL and healthcare consultation in Sri Lankan teenagers aged 13 to 18 years with abdominal pain predominant functional gastrointestinal diseases. Children with AP-FGD have significantly lower HRQoL scores for physical, emotional, social and school functioning. Approximately 28% of affected children have sought medical advice for their symptoms during previous 3 months. The main symptoms associated with healthcare consultation were abdominal bloating and vomiting. The health-related quality of life was an important determinant of healthcare consultation, more than the severity of individual symptoms.

## Abbreviations

AM: Abdominal migraine; AP-FGD: Abdominal pain predominant functional gastrointestinal disorders; FAP: Functional abdominal pain; FD: Functional dyspepsia; FGD: Functional gastrointestinal disorders; HRQoL: Health related quality of life; IBS: Irritable bowel syndrome.

## Competing interest

None of the authors have any potential competing interest.

## Authors’ contribution

ND and SR developed the initial concept and collected the data. ND analysed and wrote the initial version of the manuscript. MAB and SR contributed to the final version of the manuscript by critically analysing it. All authors are in agreement with the content of the article. All authors read and approved the final manuscript.

## Pre-publication history

The pre-publication history for this paper can be accessed here:

http://www.biomedcentral.com/1471-230X/14/150/prepub
